# T Lymphocyte Plasticity in Autoimmunity and Cancer

**DOI:** 10.1155/2015/540750

**Published:** 2015-10-25

**Authors:** Nona Janikashvili, Tinatin Chikovani, Sylvain Audia, Bernard Bonnotte, Nicolas Larmonier

**Affiliations:** ^1^INSERM U1098, Faculty of Medicine, University of Burgundy Franche-Comté, 21079 Dijon, France; ^2^Department of Microbiology and Immunology, Faculty of Medicine, Tbilisi State Medical University, 0117 Tbilisi, Georgia; ^3^Department of Internal Medicine and Clinical Immunology, University Hospital of Dijon, 21079 Dijon, France; ^4^CNRS UMR 5164, University of Bordeaux, 33076 Bordeaux, France

T lymphocytes are essential for the development and regulation of adaptive immune responses. Upon engagement of their specific antigen receptor in presence of appropriate costimulatory signals and specific cytokines, naïve CD4+ and CD8+ T cells differentiate into specific effector or suppressor subsets exhibiting distinct phenotypic characteristics and functions. These specialized T lymphocyte subsets are distinguished by a dedicated transcription factor and cytokine expression profile. Beyond T helper 1 (Th1) and T helper 2 (Th2) cells, T helper 9 (Th9), T helper 17 (Th17), T helper 22 (Th22), T follicular helper (Tfh), regulatory T cells (Treg), and Type 1 regulatory cells (Tr1) have now been recognized as distinct subsets into the CD4+ T lymphocyte compartment. Similarly, advances have also been acquired regarding CD8+ cell subset diversity (Tc1, Tc2, Tc17, and CD8reg). Although some of these polarized T cell subpopulations remain stable and hardly transdifferentiate into other subsets, evidence has been provided that others, such as Th17 or Treg cells, display variable degrees of plasticity as demonstrated by their capability to be “reprogrammed” into different suppressor or proinflammatory effector T cells. T lymphocyte plasticity depends on microenvironmental mediators such as cytokines and chemokines which vary in nature and amount during the course of autoimmune diseases or the development of cancer. A better understanding of the fundamental mechanisms and the regulation of T lymphocyte adaptive plasticity is essential for a comprehensive characterization of the pathogenesis of immune mediated disorders and cancer immunosurveillance. The possibility to interfere with the process of T cell polarization may also open new therapeutic options.

In this special issue, authors addressed topics related to the altered frequencies and functions of T lymphocyte subsets and the molecular and cellular mechanisms responsible for T cell lineage commitment, polarization, and reprogrammed adaptive responses in health and pathology. E. Ivanova and A. N. Orekhov provide an overview on the properties of T helper lymphocyte subsets and describe signaling networks, cytokines, transcription factors, and disturbances which can cause immune dysregulation, such as excessive inflammatory response in autoimmune diseases or enhanced immune tolerance to tumors. T. Caza and S. Landas summarize the epigenetic modifications of cytokine loci that determine CD4+ T cell lineage specifications and emphasize the interconnected role of the serine-threonine kinase, mammalian target of rapamycin (mTOR), and the cellular metabolism in T helper subset polarization.

T helper lymphocytes play a key role in promoting specific anticancer responses and in establishing immune memory. Tumors commonly escape from elimination by the immune system by using multiple strategies, notably the active suppression and the modulation of effector immune cells. Impaired effector T cell responses correlate with a poor prognosis in patients with solid tumors. Among numerous immune suppressive mechanisms employed by cancer, the deprivation of adenosine triphosphate (ATP) plays an important role as ATP is essential for T cell activation. Extracellular ATP concentration is maintained by the ectonucleotidase CD39 which is involved in the suppression of T lymphocyte activation by CD11c^+^ dendritic cells infiltrating tumors in the mouse lung LLC and mammary 4T1 cancer models, as described by M. Trad et al.

To suppress T effector responses, tumors can promote Treg expansion and suppressive function which correlates with a poor prognosis in cancer patients. In human, the IL-7 receptor (CD127) is often used to distinguish CD4+CD25+ Treg from activated T cells. The expression of CD127 strongly correlates with that of FoxP3, a major transcription factor that participates to the development and regulatory function of CD4+CD25+ Treg. Treg suppressive functions rely on various mechanisms involving IL-10 and TGF-*β* or cell surface ligands. The upregulated expression of TGF-*β* complexed with the latency associated peptide (LAP-TGF-*β*) on Treg provides an important functional hallmark in this subset. L. Islas-Vazquez et al. investigated the expression levels of LAP-TGF-*β* within the CD4+CD25+CD127- Treg subset in patients with stage IV lung adenocarcinoma. The data indicate that the frequencies of LAP-TGF-*β*+ Treg are elevated and the expression level of the LAP-TGF-*β* complex is significantly increased in patients with lung adenocarcinoma. Additionally, the authors report that Th17 cells and related cytokines are increased in smoking subjects and lung adenocarcinoma patients. The results suggest that smoking represent an important cause of systemic inflammation, pointing out the Th17/Treg balance as an important checkpoint in the pathophysiology of lung adenocarcinoma. Complexity of Th17 responses in the context of tumor is further discussed by L. Guery and S. Hugues. Since the infiltration of tumors by Th17 is associated with a better outcome, a strategy to promote tumor-induced Treg differentiation toward a Th17 phenotype could be a beneficial approach for immunotherapeutic interventions.

In the same line, the research article by G. Tejón et al. demonstrates that vitamin A restrains the reprogramming of* in vitro* generated Treg into IL-17 producing cells* in vivo* in an adoptive Ag-specific T cell transfer model of mouse gut inflammation. The authors additionally report that the presence of IL-2 during* in vitro* generation of Treg confers resistance to Th17 conversion and that IL-2 cooperates with retinoic acid (RA) to maintain Foxp3 expression following stimulation under Th17-polarizing conditions. Of importance, gut microbiome significantly shapes T lymphocyte dynamics in an inflammatory condition such as IBD, as extensively reviewed by C. Larmonier et al. Therefore, Th17 and Treg stability and plasticity are regulated not only by the signals they receive during their generation but also by their local microenvironment.

The acute phase response also represents an example of peripheral immune modulation. In the context of systemic inflammation, some proteins, among which is *α*1-antitrypsin, exert tolerogenic activities. The topic reviewed by B. Baranovski et al. underlines that *α*1-antitrypsin can indirectly inhibit T cell responses, by targeting other immune cells. For instance, *α*1-antitrypsin maintain the semimature state of DC which promotes Treg differentiation. This process appears pivotal during experimental autoimmune encephalomyelitis, rheumatoid arthritis, ulcerative colitis, and type 1 diabetes.

